# Insight into the origin of chikungunya virus in Malaysian non-human primates via sequence analysis

**DOI:** 10.1016/j.heliyon.2019.e02682

**Published:** 2019-12-07

**Authors:** O. Suhana, W.A. Nazni, Y. Apandi, H. Farah, H.L. Lee, M. Sofian-Azirun

**Affiliations:** a Institute of Biological Sciences, Faculty of Science, University of Malaya, 50603 Kuala Lumpur, Malaysia; b Medical Entomology Unit and WHO Collaborating Centre for Vectors, Institute for Medical Research, Jalan Pahang, 50588 Kuala Lumpur, Malaysia; c Virology Unit, Institute for Medical Research, Jalan Pahang, 50588 Kuala Lumpur, Malaysia

**Keywords:** Genetics, Virology, Chikungunya, Phylogenetic analysis, Non-human primates, Malaysia

## Abstract

Chikungunya virus (CHIKV) is maintained in the sylvatic cycle in West Africa and is transmitted by *Aedes* mosquito species to monkeys. In 2006, four verified CHIKV isolates were obtained during a survey of arboviruses in monkeys (*Macaca fascicularis*) in Pahang state, Peninsular Malaysia. RNA was extracted from the CHIKV isolates and used in reverse transcription polymerase chain reactions (RT-PCR) to amplify PCR fragments for sequencing. Nucleic acid primers were designed to generate overlapping PCR fragments that covered the whole viral sequence. A total of 11,238 base pairs (bp) corresponding to open reading frames (ORFs) from our isolates and 47 other registered isolates in the National Center for Biotechnology Information (NCBI) were used to elucidate sequences, amino acids, and phylogenetic relationships and to estimate divergence times by using MEGA 7.0 and the Bayesian Markov chain Monte Carlo method. Phylogenetic analysis revealed that all CHIKV isolates could be classified into the Asian genotype and clustered with Bagan Panchor clades, which are associated with the chikungunya outbreak reported in 2006, with sequence and amino acid similarities of 99.9% and 99.7%, respectively. Minor amino acid differences were found between human and non-human primate isolates. Amino acid analysis showed a unique amino acid at position 221 in the nsP1region, at which a glycine (G) was found only in monkey isolates, whereas arginine (R) was found at the same position only in human isolates. The time to the most recent common ancestor (MRCA) estimation indicated that CHIKV probably started to diverge from human to non-human primates in approximately 2004 in Malaysia. The results suggested that CHIKV in non-human primates probably resulted from the spillover of the virus from humans. The study will be helpful in understanding the movement and evolution of CHIKV in Malaysia and globally.

## 1 Introduction

Chikungunya virus (CHIKV) belongs to the genus *Alphavirus* in the family *Togaviridae*. It is a mosquito-borne infectious agent and was first detected in 1952 in Africa at the Makonde Plateau (border area between Tanzania and Mozambique). CHIKV has a positive, single-stranded RNA genome, an approximately 700 nm diameter capsid and a phospholipid envelope. The genome of CHIKV consists of 5′ cap-nsP1-nsP2-nsP3-nsP4-(Junction region)-C-E3-E2-6K-E1-poly (A) 3’ [1]. The first complete genome of CHIKV was reported in 2002, known as strain S27 (African prototype), using RT-PCR techniques [1].

Phylogenetic analyses have shown that there are three major CHIKV genotypes, namely, Asian, West African, and East Central South Africa (ECSA), with the recent Indian Ocean outbreak and Caribbean outbreak forming monophyletic clades that are descendants of the ECSA and Asian genotypes, respectively [2, 3]. In Africa, the virus is maintained in a sylvatic cycle involving wild primates and many species of *Aedes*, especially *Ae. furcifer* and *Ae. africanus* [4], but in Asia, the virus is generally believed to be largely maintained in a mosquito-human-mosquito cycle with the urban anthropophilic *Ae. aegypti* as the primary transmission vector [5].

Strain S27 was the first CHIKV isolated from human serum in Tanzania in 1953, and *Ae. aegypti* was identified as the main vector [6]. Neutralizing antibodies to CHIKV were reported in human adults in West Malaysia during 1965–1969, especially among those living in states bordering Thailand. CHIKV-neutralizing antibodies were also detected in wild monkeys, pigs, and chickens in Malaysia [7], which suggested that the CHIKV sylvatic transmission cycle may exist in this country, even though viable or live CHIKV has never been isolated from Asian monkeys [7].

In a surveillance to identify potential pandemic threats and detect the presence of arbovirus in non-human primates (*Macaca fascicularis*), Apandi et al. (2009) isolated and characterized CHIKV from four sera of non-human primates [8]. However, the study focused only on the E1 and nsP1 regions. As a continuation of Apandi et al. (2009), the aims of the present study are to generate and characterize the full genome sequence of CHIKV infecting non-human primates and subsequently construct a phylogenetic tree using isolation times as calibration points to establish the date of introduction of CHIKV to non-human primates and the rate evolution patterns of CHIKV in Malaysia. A comprehensive phylogenetic study of CHIKV requires the analysis of complete ORF sequences for both non-structural and structural polyproteins [3].

## 2 Materials and methods

### 2.1 Ethics statement and origin of samples

Viral isolation was conducted on serum samples from monkeys trapped by the Department of Wildlife and National Parks Peninsular Malaysia (PERHILITAN). All study protocols and experiments were performed according to regulation and were approved by the Animal Care & Use Committee (ACUC), Institute for Medical Research, Kuala Lumpur and Medical Research Ethics Committee, National Institutes of Health, Ministry of Health, Malaysia (approval number: NMRR-08-295-1450). All procedures for handling non-human primates, including injections or blood withdrawals, were performed under anaesthesia with intramuscular injection of ketamine hydrochloride (10 mg/kg body weight) and in accordance with established guidelines. After sera were drawn, the monkeys were returned to PERHILITAN and released back to their natural habitat. No monkey was sacrificed in this study.

The viral strains used in this study were isolated from long-tailed macaques (*Macaca fascicularis*) trapped in Kuala Lipis, Pahang, in 2008 in Serunai Mela Village [4°7.0′N, 102°11.9′E], Sungai Ular [4°15.7′N, 102°4.8′E] and a forested area [4°12.673′N, 101°53.127′E] from August 2007 to January 2008.

### 2.2 CHIKV propagation

CHIKV was propagated in the Vero cell line (African green monkey kidney) as described in Apandi et al. [6]. Briefly, the second passage of the virus propagated in Vero cells at 37 °C under 5% CO_2_ was maintained in Hank's MEM medium supplemented with 2% foetal bovine serum (FBS), 1% penicillin–streptomycin (pen-strep), 1 M HEPES and 7.5% sodium bicarbonate. The development of cytopathic effects (CPEs) was observed daily, and CPEs appeared on day 4. Four viral isolates labelled M125, M127, M128, and M129 were used in the subsequent studies as reported by Apandi et al. [6]. The identification of the CHIKV isolates was performed by using RT-PCR with specific primers targeting the E1 gene as reported by Hasebe et al. [7].

### 2.3 Reverse transcriptase-polymerase chain reaction (RT-PCR)

Viral RNA was extracted from 140 μl supernatant of Vero cell culture infected with CHIKV using the QIAamp® Viral RNA Mini Kit from Qiagen (Hilden, Germany). Five microlitres of extracted viral RNA was converted to cDNA using 2 μl of 10 μM reverse primers (Table 1) followed by incubation at 70 °C for 10 min. Then, RT master mix containing 2.0 μl of 5X Moloney Murine Leukaemia virus (M-MLV) reverse transcriptase buffer (Promega Madison, WI, USA), 0.5 μl of 100 units AMV reverse transcriptase (Promega Madison, WI, USA) and 0.5 mM dNTPs (Promega Madison, WI, USA) was added to cDNA. The mixtures were then incubated at 42 °C for 60 min followed by 10 min at 70 °C to stop the reaction.

**Table 1:** Set of primers used in RT-PCR.

Primer	Gene Position	Sequence 5′-3′	Annealing Temperature (^o^C)
1F1	1–21	atggctgcktgagacacacgt	62.8
1R525	525–548	cgtgtacagcatagacgtcttggt	
1F409	409–432	catytctggaaagatcggggactt	62.5
1R819	819–842	taagtagcgtgcggctttccgggt	
1F804	804–825	tagggtcaacgctttacccgga	63.9
1R1375	1375–1398	tggattgactgggtatcaggcctc	
1F1311	1311–1334	aaagaacactaacctgctgctgtc	63.3
1R1867	1867–1890	aggactcggccatcgtacgcttcg	
1F1800	1800–1822	tgatccacgctttagcggagcaa	63.6
1R2375	2375–2398	caagagcagcgaatctaccgtacg	
1F2320	2320–2340	ctgccaagaaatcagcaccga	61.5
1R2874	2874–2896	tgatgcatagagtgggttttcgt	
1F2801	2801–2824	cacgaggtcatgacagcagccgca	62.7
1R3365	3365–3388	ttctagaatggacgctgcctcagg	
1F3303	3303–3324	ctgtgtattacgcggataacca	61.2
1R3822	3822–3843	gagccacccggtttgagcagtc	
1F3808	3808–3829	gggtgactcactgagactgctc	62.5
1R4328	4328–4351	gacttctcgataggcagccgccaa	
1F4320	4320–4341	accgggaattggcggctgccta	64.7
1R4877	4877–4900	tcgggtgacgcgttcaggagtcat	
1F4800	4800–4822	tcaggcagaaatgcccggtggat	64.6
1R5368	5368–5389	tacggccggacacagctctgct	
1F5218	5218–5241	cataatcggaaaccttgcggccgt	64.4
1R5832	5832–5855	acctgcttctattggccatggacg	
1F5818	5818–5838	actccaggagagtgcgtccat	61.9
1R6317	6317–6340	cacgttgaatactgctgagtccaa	
1F6303	6303–6326	gggaattacccactttggactcag	61.3
1R6845	6845–6865	cagggagtgatccactcccaa	
1F6700	6700–6720	atttgacatgtctgccgagga	61.7
1R7222	7222–7243	tagcgggtccgccactctgcaa	
1F7240	7204–7226	tactgtgacaggaacagcttgca	62.7
1R7719	7719–7714	gttgaggtaccgcgcattgtc	
1F7716	7716–7737	actgacaatgcgcgcggtacct	64.5
1R8275	8275–8296	tcaccaccgagagggctgtacg	
1F8201	8201–8221	acagcggtagaccgatcttcg	63.7
1R8741	8741–8764	gcatgtgattgtccatgtaacgca	
1F8721	8721–8741	tagccatgattggaccaagct	62.3
1R9213	9213–9236	tgattggtgaccgcggcatggcat	
1F9204	9204–9227	ggttgatcaatgccatgccgcggt	64.3
1R9741	9741–9764	acggtagctcctggtgttcagttcg	
1F9702	9702–9722	gatgtgcatgtgtgcacgacg	63.5
1R10236	10236–10259	cacatgaatgggtagacgccggtg	
1F10102	10102–10122	actttggagccaacgctatcg	62.9
1R10677	10677–10700	gcctgagagtacggcacgtgcacc	
1F10600	10600–10620	atccaaagtcgcacgcctgag	64.3
1R11179	11179–11199	aatgtcttggaccccgagggt	
1F11113	11113–11136	gtacactgtgcagccgagtgccat	60.0
1R11811	11811–11834	agccactttgaacatctcctacgt	
1F11200	11200–11223	tccgctacggcgatgtcatgggtg	59.3
1R11811	11811–11834	agccactttgaacatctcctacgt	
1F11313	11313–11335	acttgacaactaggtacgaaggt	57.3
1R11811	11811–11834	agccactttgaacatctcctacgt	

Twenty microlitres of PCR mixture containing 5 μl of cDNA, 12.5 μl of AccessQuick™ Master Mix (Promega Madison WI, USA) and 1.0 μl of reverse and forward primers was prepared (Table 1). The mixture was then incubated at 94 °C for 3 min, 94 °C for 30 min, 62.8 °C for 1 min and 72 °C for 10 min. The PCR products were visualized by 2% agarose gel electrophoresis in 1X TBE buffer containing 0.5 μg/ml ethidium bromide. DNA was extracted from agarose gels using the Gel Extraction Kit (Qiagen, Hilden, Germany). The 5′ and 3′ ends of the genomes were amplified using 5′ and 3’ rapid amplification of cDNA ends (RACE) Kits (Invitrogen, Carlsbad, CA).

### 2.4 Sequencing and phylogenetic analysis

All amplicons were sequenced in both directions by using the PCR primers in Table 1. Sequencing was performed by using the Big Dye Cycle Sequencing Kit (version 3.0) and an ABI377 automated DNA sequencer (Applied Biosystems, Foster City, USA). The SeqMan software module in the Lasergene suite of programs (DNASTAR, Madison, USA) was used to format the nucleotide sequences. All sequences for each virus were aligned and assembled using Megalign software and the SeqMan module in the Lasergene suite of programs (DNASTAR, Madison, USA). Analysis of nucleotide and deduced amino acid sequence identities was performed using GeneDoc V 2.7 [8] and the Lasergene suite of programs (DNASTAR, Madison, USA).

A total of 47 reference sequences of CHIKV were retrieved from the GenBank database with sampling dates ranging from 1953-2014. The best fit model of nucleotide substitution was determined using MODELTEST 3.7 (Posada & Crandall, 1998). Phylogenetic trees were reconstructed by using MEGA 7.0 [9]. Maximum likelihood (ML) in the Kimura 2 parameter model with gamma distribution and invariant sites was adopted. The nodal reliability of ML was assessed by bootstrapping (BS) with 1,000 replicates.

Coalescent trees were also reconstructed via Bayesian analysis, and the time of the most recent common ancestor (MRCA) was calculated using BEAST package v.1.8.3 with Markov chain Monte Carlo algorithms (MCMC) [10]. BEAUTI V.1.8.3 was used to create an input file for BEAST. The data were analysed using the general time reversible (GTR) with gamma distribution (G) as selected by MODELTEST 3.7 [11]. The GTR approach is considered the most general, neutral, and independent approach that accounts for finite sites and is time reversible [12]. “G” represents the gamma distribution, which is a two-parameter family with a continuous probability distribution. Uncorrelated lognormal relaxed molecular clock was used for analysis [13] based on the fact that no a priori correlation existed between a lineage's rate of evolution. MCMC analysis was run for 30,000,00 chains, and the data trees were sampled every 3,000 generations. Convergence of the MCMC sample on the posterior distribution was defined at an effective sample size (ESS) value >200, which was calculated by the Tracer v.1.4 program. The maximum clade credibility (MCC) tree was constructed using TreeAnnotator v.1.8.3 [10]. The convergence of parameters was verified with Tracer v.1.6.0, and the uncertainties were addressed at 95% highest probability density (HPD) intervals. The final tree was displayed with FigTree v.1.4.2 [10].

## 3 Results and discussion

### 3.1 Viral isolation and identification

Infected Vero cells were harvested upon the appearance of prominent CPEs 4 days post-infection. Isolates were confirmed to be CHIKV positive by RT-PCR. The primer pair used for confirmation was based on the E1 targeted region [7] for detection of CHIKV. Sequence analysis showed that they exhibited high sequence identity with the CHIKV human isolate from Bagan Panchor, Perak, Malaysia. The presence of CHIKV was confirmed in all studied samples as previously described by Apandi et al. (2009) [6] by PCR and sequencing.

### 3.2 Genome organization and sequence analysis

The full coding sequence of the four isolates from Malaysian non-human primates was 11,837 nucleotides. The sequences were deposited in the GenBank database with the following accession numbers: KM923917, KM923918, KM923919, and KM923920. The positive single-strand RNA genome was divided into three parts: the 5′ noncoding area that was 76 nucleotides long, the 3’ noncoding area that was 538 nucleotides long, including an internal poly (A) region of 3′ NTR, and the coding region. The calculated base composition was A (29.5%), C (24.4%), G (25.4%), and T (20.6%). The CHIKV reference S27 was used, which contains two independent ORFs, a non-structural region with a length of 7,422 nucleotides encoding the non-structural proteins (nsP1, nsP2, nsP3 and nsP4) and the structural region with 3,732 nucleotides encoding structural proteins (C, E3, E2, 6K and E1). The untranslated junction region between the two coding areas is 61 nucleotides long.

### 3.3 Genotypic characterization and comparison of non-human CHIKV isolates with Asian genotype human isolates

Due to the lack of a 5′UTR and the rapid sequence divergence of the 3′UTR in other sequences in GenBank, only ORFs were adopted for subsequent analyses. Sequence analysis revealed that the four non-human primate isolates were the Asian genotype and closely related to the Bagan Panchor 2006 isolate (Tables 2 and 3). Comparison between the four isolates of Malaysian non-human primate (M125, M127, M128, and M129) and human Bagan Panchor CHIKV isolates (FN295483, FN295484, EU703759, EU703760, EU703761, and EU703762) showed a high percentage of similarity ranging from 99.7-99.9% (Tables 2 and 3). Furthermore, the four non-human primate isolates (M125, M127, M128, and M129) showed nucleotide identity of 94.4% and amino acid identity of 97.1% with strain S27 in all ORFs (summary of nucleotide and amino acid identities are shown in Tables 2 and 3.

**Table 2:** Nucleotide identity and amino acid identity of non-human primates (M125, M127, M128, M129) in ORF compared with other isolates. Each strains was labeled using following format. “Accession number”, “Isolate”, “Country of origin”, and “Year isolated”.

Strain	Nucleotide identity (%)	Amino acid identity (%)
EU703759(MY002IMR)-Malaysia-2006	99.9	99.9
EU703760(MY003IMR)-Malaysia-2006	99.9	99.9
EU703761(MY019IMR)-Malaysia-2006	99.8	99.7
EU703762(MY021IMR)-Malaysia-2006	99.8	99.7
FN295483 (MY.37348)-Malaysia-2006	99.7	99.7
FN295484(MY.37350)-Malaysia-2006	99.7	99.7
KF872195(Leiv.Chik)-Russia-2013	99.2	99.5
KF872195(LEIV-CHIKV/Moscow/1/2013)-Moscow-2013	99.2	99.5
HE806461(NC_2011-568)-NewCaledonia-2011	99.3	99.6
FJ807897(0706aTw)-Indonesia-2007	99.4	99.4
CXNT01000000(CK12-921)-Philippines-2012	99.2	99.3
KF318729(chik-sy)-China-2012	99.1–99.2	99.4
CWIF01000000(CK12-674)-Philippines-2012	99.1–99.2	99.4
CWHX01000000(CK12-559)-Philippines-2012	99.1	99.4
CWIC01000000(CK12-708)-Philippines-2012	99.1	99.3
KJ451622(3807)-Micronesia-2013	99.1	99.4
CWHZ01000000(CK12-882)-Philippines-2012	99.1	99.3
AB860301(CHIKV-13-112A)-Philippines-2013	99.1	99.4
KX262991(H20235)-StMartin-2013	99.1	99.3
KJ451624(99659)-BritishVirginIsland-2014	99.1	99.3
FJ000068(IND-KA51)-India-2006	99.1	99.3
KT327167(TA0006)-Mexico-2013	99.1	99.3
KT327165(CH0072)-Mexico-2013	99.1	99.3
KT327164(CH0045)-Mexico-2013	99.1	99.3
EF027141(IND-73-MH5)-India-1973	97.5	98.6
HM045788(PO731460)-India-1973	97.5	98.6
HM045813(Gibbs263)-India-1963	97.9	98.7
HM045803(I-634029)-India-1963	97.9	98.8
EF027140(IND-63-WB1)-India-1963	97.7	98.7
HM045810(TH35)-Thailand-1958	98.2	99.0
EF452493(AF15561)-Thailand-1962	98.3	99.1
HM045814(1455)-Thailand-1975	98.6	99.1
HM045802(K0146)-Thailand-1995	98.2	99.0
HM045787(SV0444)-Thailand-1995	98.2	98.9
HM045796(CO392)-Thailand-1995	98.3	99.0
HM045789(6441)-Thailand-1988	98.5	98.8
HM045800(Hu.85.NR.001)-Philippines-1985	98.8	99.1
HM045790(PhH15483)-Philippines-1985	98.8	99.1
HM045791(JKT23574)-Indonesia-1983	98.9	99.2
HM045797(RSU1)-Indonesia-1985	98.8	99.1
AF369024(S27)-Tanzania-1953	94.4	97.1
KJ451623(3462)-Micronesia-2013	99.1	99.4
EU564334(TM25)-Mauritius-2006	93.7	96.8

**Table 3:** Percentage identity of sequences (above the diagonal) and amino acid sequence (below the diagonal) between human and non-human primate (nucleotide region 55–11889).

	Strain		1	2	3	4	5	6	7	8	9	10	11	12	13	14	15	16	17	18	19	20	21
Genotype	S27	1	-	94.0	94.0	94.0	94.0	93.9	93.9	94.1	94.1	94.0	94.0	94.0	94.1	94.5	94.2	95.2	97.1	97.1	97.1	85.0	73.9
Non-Human primates isolate	M125	2	96.4	-	99.9	100.0	100.0	99.7	99.7	99.9	99.9	99.8	99.8	99.3	98.1	98.5	98.2	97.7	93.5	93.6	93.6	83.7	73.5
	M127	3	96.4	100.0	-	100.0	100.0	99.7	99.7	99.9	99.9	99.8	99.8	99.3	98.1	98.5	98.2	97.7	93.5	93.6	93.6	83.7	73.5
	M128	4	96.4	100.0	100.0	-	99.9	99.7	99.7	99.9	99.9	99.8	99.8	99.3	98.1	98.5	98.2	97.7	93.5	93.6	93.6	83.7	73.5
	M129	5	96.4	100.0	100.0	100.0	-	99.7	99.7	99.9	99.9	99.8	99.8	99.3	98.1	98.5	98.2	97.7	93.5	93.6	93.6	83.7	73.5
Asian	FN295483	6	96.5	99.9	99.9	99.9	99.9	-	100.0	99.7	99.7	99.6	99.7	99.4	98.0	98.4	98.1	97.5	93.3	93.5	93.5	83.5	73.5
	FN295484	7	96.5	99.9	99.9	99.9	99.9	100.0	-	99.7	99.7	99.6	99.7	99.4	97.9	98.3	98.0	97.5	93.3	93.4	93.4	83.5	73.5
	EU703759	8	96.5	99.9	99.9	99.9	99.9	100.0	100.0	-	100.0	99.9	99.9	99.3	98.1	98.5	98.2	97.7	93.5	93.6	93.6	83.7	73.6
	EU703760	9	96.5	99.9	99.9	99.9	99.9	100.0	100.0	100.0	-	99.9	99.9	99.3	98.1	98.5	98.2	97.7	93.5	93.6	93.6	83.7	73.6
	EU703761	10	96.4	99.8	99.8	99.8	99.8	99.9	99.9	99.9	99.9	-	99.8	99.2	98.1	98.4	98.1	97.6	93.4	93.5	93.5	83.6	73.5
	EU703762	11	96.4	99.8	99.8	99.8	99.8	99.8	99.8	99.8	99.8	99.8	-	99.2	98.0	98.5	98.1	97.7	93.4	93.5	93.5	83.6	73.5
	FJ807897	12	96.5	99.8	99.8	99.8	99.8	99.8	99.8	99.8	99.8	99.7	99.6	-	98.0	98.4	98.1	97.5	93.4	93.5	93.5	83.7	73.5
	HM045800	13	97.0	99.1	99.1	99.1	99.1	99.1	99.1	99.2	99.2	99.1	99.0	99.8	-	98.7	99.2	97.7	93.6	93.8	93.7	83.6	73.5
	HM045789	14	97.1	99.0	99.0	99.0	99.0	99.0	99.0	99.1	99.1	99.0	98.9	99.1	99.8	-	99.0	98.2	93.9	94.0	94.0	83.9	73.7
	HM045802	15	96.8	98.7	98.7	98.7	98.7	98.7	98.7	98.8	98.8	98.7	98.6	98.8	99.4	99.4	-	97.9	93.6	93.8	93.8	83.9	73.9
	EF027140	16	97.4	98.2	98.2	98.2	98.2	98.3	98.3	98.3	98.3	98.2	98.2	98.3	99.0	98.9	98.6	-	94.6	94.7	94.7	84.2	73.9
ECSA	FN295487	17	98.3	96.1	96.1	96.1	96.1	96.2	96.2	96.2	96.2	96.1	96.1	96.2	96.7	96.7	96.5	97.0	-	99.8	99.8	84.9	74.0
	FJ807896	18	98.3	96.2	96.2	96.2	96.2	96.2	96.2	96.2	96.2	96.2	96.1	96.2	96.7	96.7	96.5	97.0	99.9	-	99.9	84.9	74.0
	EF027138	19	98.3	96.1	96.1	96.1	96.1	96.2	96.2	96.2	96.2	96.2	96.0	96.2	96.6	96.6	96.4	99.9	99.9	99.9	-	84.9	74.0
WA	AY726732	20	93.7	91.9	91.9	91.9	91.9	91.9	92.0	92.0	92.0	91.9	91.8	92.1	92.4	92.5	92.2	92.9	93.5	93.5	93.5	-	74.3
ONN	SG650	21	84.9	84.3	84.3	84.3	84.3	84.3	84.4	84.3	84.3	84.2	84.2	84.4	84.8	84.7	84.4	84.7	85.1	85.2	85.2	86.2	-

This study identified several amino acid changes in various regions of CHIKV isolates, with the highest amino acid difference found between Malaysian non-human primates and strain S27 in E3 (7.8%), followed by nsP3 (5.5%), E2 (5.0%), 6K (4.9%), nsP1 (2.6%), C (1.9%), E1 (1.8%), and nsP2 (1.2%) (Table 4). There was a relatively higher level of mutation found in the structural protein region than in the non-structural region. This may be because the non-structural region encodes functional proteins that play a role in CHIKV replication [14].

**Table 4:** Amino acid differences between the non-structural and structural region sequences of CHIKV isolated from Malaysian non-human primates, other Malaysia Asian genotype isolates and S27.

Protein	Regions	Amino acid differences between non-human primate isolates and S27	Amino acid differences between non-human primates and Malaysia Asian genotype isolates.
Non-structural region	nsP1	S3P, S34P, V172L, **G221R**, K234E, M253K, L383M, G454S, R473S, A478T, I481T, N486D, Q491R, H507R	**G221R**
	nsP2	L16P, S218T, L273Q, M338K, V466M, V486I, Y642C, N643S, I756V, S768N,	
	nsP3	T77S, I176V, V213M, H217Y, N283S, S326P, R332Q, V334A, A349V E352K, T353I, V376I, T381S, I382A, I383T, I413T, Q434L, A437V, I449M, R452Q, I457T, T458A, V459T, P461S, P471S, D483N, D484E, *524R	VHTLPST 376–382 Del
	nsP4	L42A, K85R, A90S, V101T, R235Q, R271K, D280E, A366T, T514I, I555V A582V, I604V	
Structural region	C	K37Q, R78Q, M81T, V93A	A55V*
	E3	T23I, K33E, S44R, R60H	
	E2	I2T, H5N, K57G, M74I, E79G, G118S, R149K, A157V, T160N, M181L, D205G, S207N, T211I, L255I, R267M, N299S, I317V, R318V, T344A, V384M	
	E1	S72N, T98A, A145T, E211K, S225A, S304P, A322V, P397L	

Amino acid differences reported following the alignment of sequences obtained from non-human primate isolates (M125, M17, M128, and M129). The first amino acid named was found in the non-human primate isolate, while the second amino acid was found in S27 and Malaysia Asian genotype strains. Differences at sites between non-human primate and human isolates are underlined. (Malaysia Asian genotype isolates consist of FN295483, FN295484, EU703759, EU703760, EU703761, and EU703762).

The serum was cultured before PCR to confirm the presence of infectious or viable virus circulating in the environment and for viral stock since serum from non-human primates was limited [15]; the detection of CHIKV RNA by PCR alone is not sufficient to confirm the presence of infectious virus because PCR can detect inactivated viral RNA even if it was stored for many weeks at 28 °C. This is the first discovery of viable CHIKV in wild monkeys. Previously, the detection of chikungunya was by CHIKV-neutralizing antibodies in wild monkeys [16].

It was challenging to detect and amplify the target DNA in this study. Several sets of primers were used but resulted in unsuccessful amplifications. Initially, a specific virus was targeted by PCR with primers 188–222, 012-011, OL68-EVP2 and 189–222, which were reported by Oberste et al. (2002) [17] to detect simian retrovirus in non-human primates. Unfortunately, this resulted in negative amplifications. In 1986, Rudnick and Lim [17] reported the presence of arbovirus, especially dengue virus, in non-human primates in Malaysia by using universal primers and flavivirus universal primers [18]. The primer sets were applied in our study but were unable to amplify target DNA via PCR. This may be due to multiple mismatches in the priming sites that caused a reduction in sensitivity [19].

As shown in Table 4, a glycine (G) residue was found at position 221 of the nsP1 region in non-human primate isolates and was substituted with an arginine (R) in the same region in human isolates. One of the possible reasons for this mutation may be due to *in vitro* adaptation. However, we believe that the change in amino acids is not due to serial dilution but to the adaptation of CHIKV RNA to a specific host. Based on 50 sequences of CHIKV, position 221 is highly conserved (Table 5), while the mutation can be observed only in amino acid sequences of non-human primates.

**Table 5:** Alignment of CHIKV nsP1 amino acid (209–233) sequences.

Virus-Acession no.- country- year	nsP1 sequence (nucleotides 209–233)
	209 221 233
CHIKV-KM923920(M129)-Malaysia-2008	NIGLCSTDLTEG**G**RGKLSIMR
CHIKV-KM923918(M127)-Malaysia-2008	NIGLCSTDLTEG**G**RGKLSIMR
CHIKV-KM923919(M128)-Malaysia-2008	NIGLCSTDLTEG**G**RGKLSIMR
CHIKV-KM923917(M125)-Malaysia-2008	NIGLCSTDLTEG**G**RGKLSIMR
CHIKV- HM045803(I-634029)-India-1963	NIGLCSTDLTEGRRGKLSIMR
CHIKV- HM045813(Gibbs263)-India-1963	NIGLCSTDLTEGRRGKLSIMR
CHIKV- EF027140(IND-63-WB1)-India-1963	NIGLCSTDLTEGRRGKLSIMR
CHIKV- HM045788(PO731460)-India-1973	NIGLCSTDLTEGRRGKLSIMR
CHIKV- EF027141(IND-73-MH5)-India-1973	NIGLCSTDLTEGRRGKLSIMR
CHIKV- HM045810(TH35)-Thailand-1958	NIGLCSTDLTEGRLGKLSIMR
CHIKV- EF452493(AF15561)-Thailand-1962	NIGLCSTDLTEGRRGKLSIMR
CHIKV- HM045814(1455)-Thailand-1975	NIGLCSTDLTEGRRGKLSIMR
CHIKV- HM045789(6441)-Thailand-1988	NIGLCSTDLTEGRRGKLSIMR
CHIKV- HM045787(SV0444)-Thailand-1995	NIGLCSTDLTEGRRGKLSIMR
CHIKV- HM045802(K0146)-Thailand-1995	NIGLCSTDLTEGRRGKLSIMR
CHIKV- HM045796(CO392)-Thailand-1995	NIGLCSTDLTEGRRGKLSIMR
CHIKV- HM045791(JKT23574)-Indonesia-1983	NIGLCSTDLTEGRRGKLSIMR
CHIKV- HM045797(RSU1)-Indonesia-1985	NIGLCSTDLTEGRRGKLSIMR
CHIKV- HM045790(PhH15483)-Philippines-1985	NIGLCSTDLTEGRRGKLSIMR
CHIKV- HM045800(Hu.85.NR.001)-Philippines-1985	NIGLCSTDLTEGRRGKLSIMR
CHIKV- FN295483(MY.37348)-Malaysia-2006	NIGLCSTDLTEGRRGKLSIMR
CHIKV- FN295484(MY.37350)-Malaysia-2006	NIGLCSTDLTEGRRGKLSIMR
CHIKV- HE806461(NC 2011-568)-NewCaledonia-2011	NIGLCSTDLTEGRRGKLSIMR
CHIKV- KF872195(Leiv.Chik)-Russia-2013	NIGLCSTDLTEGRRGKLSIMR
CHIKV- CWJV01000000(CK12-148)-Philippines-2012	NIGLCSTDLTEGRRGKLSIMR
CHIKV- CWIC01000000(CK12-708)-Philippines-2012	NIGLCSTDLTEGRRGKLSIMR
CHIKV- CXNV01000000(CK12-709)-Philippines-2012	NIGLCSTDLTEGRRGKLSIMR
CHIKV- CWHZ01000000(CK12-882)-Philippines-2012	NIGLCSTDLTEGRRGKLSIMR
CHIKV- CWIE01000000(CK12-884)-Philippines-2012	NIGLCSTDLTEGRRGKLSIMR
CHIKV- CWIJ01000000(CK12-906)-Philippines-2012	NIGLCSTDLTEGRRGKLSIMR
CHIKV- CWHX01000000(CK12-559)-Philippines-2012	NIGLCSTDLTEGRRGKLSIMR
CHIKV- CXNT01000000(CK12-921)-Philippines-2012	NIGLCSTDLTEGRRGKLSIMR
CHIKV- CWIF01000000(CK12-674)-Philippines-2012	NIGLCSTDLTEGRRGKLSIMR
CHIKV- KF318729(chik-sy)-China-2012	NIGLCSTDLTEGRRGKLSIMR
CHIKV- KJ451623(3462)-Micronesia-2013	NIGLCSTDLTEGRRGKLSIMR
CHIKV- EU703759(MY002IMR)-Malaysia-2006	NIGLCSTDLTEGRRGKLSIMR
CHIKV- EU703760(MY003IMR)-Malaysia-2006	NIGLCSTDLTEGRRGKLSIMR
CHIKV- EU703761(MY019IMR)-Malaysia-2006	NIGLCSTDLTEGRRGKLSIMR
CHIKV- EU703762(MY021IMR)-Malaysia-2006	NIGLCSTDLTEGRRGKLSIMR
CHIKV- AF369024(S27)-Tanzania-1953	NIGLCSTDLTEGRRGKLSIMR
CHIKV- AB455493(SL11131)-India-2006	NIGLCSTDLTEGRRGKLSIMR
CHIKV- FJ807897(0706aTw)-Indonesia-2007	NIGLCSTDLTEGRRGKLSIMR
CHIKV- EU564334(TM25)-Mauritius-2006	NIGLCSTDLTEGRRGKLSIMR
CHIKV- KJ451622(3807)-Micronesia-2013	NIGLCSTDLTEGRRGKLSIMR
CHIKV- KJ451624(99659)-British Virgin Islands-2014	NIGLCSTDLTEGRRGKLSIMR
CHIKV- FJ000068(IND-KA51)-India-2006	NIGLCSTDLTEGRRGKLSIMR
CHIKV- KT327164(CH0045)-Mexico-2013	NIGLCSTDLTEGRRGKLSIMR
CHIKV- KT327167(TA0006)-Mexico-2013	NIGLCSTDLTEGRRGKLSIMR
CHIKV- KT327165(CH0072)-Mexico-2013	NIGLCSTDLTEGRRGKLSIMR
CHIKV- AB860301(CHIKV-13-112A)-Philippines-2013	NIGLCSTDLTEGRRGKLSIMR
CHIKV- KX262991(H20235)-StMartin-2013	NIGLCSTDLTEGRRGKLSIMR

Due to a lack of information regarding the structure of nsP1, the role of the mutation in amino acid position 221 in the structure of nsP1 is unclear. It was postulated that these disordered regions may participate in specific interactions with other proteins, nucleic acids or membranes [20]. The nsP1 proteins have guanine-7-methyltransferase and guanylyltransferase activities, which are essential for capping in the cap methylation of viral RNA by a mechanism that is distinct from the host RNA capping mechanism [21, 22, 23, 24]. The specific mutation may affect the binding of enzymatic substrates at amino acid 310 of nsP1 [25]. According to Burrack et al. (2014) [26], the mutation in nsP1 can modulate the function of nsP1 in RNA synthesis and/or capping activities. The latter can increase the effectiveness of interferon (IFN) that stimulates antiviral effectors. This happens during the acute stage of CHIKV infection, which is one to six days after infection [27]. Interestingly, these effects appear to be operative in skeletal muscle tissues but not in joint-associated tissues, and this may be one of the reasons why non-human primates do not experience arthralgia or joint pain in the subacute and chronic phases [28].

**Fig. 1:**
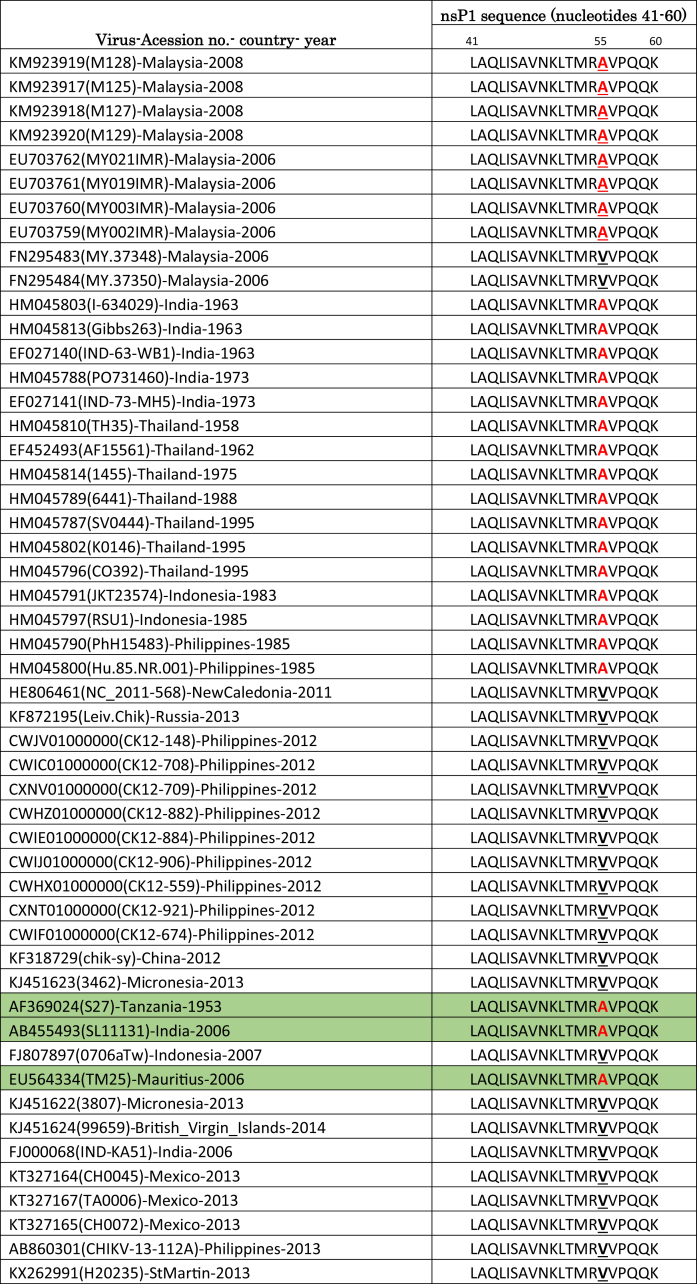
Alignment of CHIKV Capsid amino acid (41-60) sequences.

The mutation in amino acid A55V in 2 Malaysian strains (FN295483 and FN295484) can be observed in other CHIKV isolates (Fig. 1). The 55A mutation can be observed in the first CHIKV isolate (S27) from 1953 to 2006. The reported isolate, identified with this mutation from 2006 to 2014, switched to 55V (Fig. 1). The mutation has not been clearly elucidated. The capsid protein of Sindbis virus (SINV) is divided into three regions: region I, with residues 1–80; region II, with residues 81–113; and region III, with residues 114–264. Regions I and II are part of the N-terminal domain of the capsid protein and are involved in encapsidation of genomic RNA [29]. The N-terminal domain is less conserved and is intrinsically disordered with a high degree of positive charge, which is responsible for binding to the RNA genome, shutting host transcription off and the dimerization of the capsid protein [30, 31]. Region III is part of the C-terminal domain, which is responsible for the serine protease activity of the capsid protein. The capsid protein has cis-proteolytic activity that cleaves itself from the nascent structural polyprotein precursor [32, 33, 34, 35]. The C-terminal protease domain of the Alphavirus capsid protein is highly conserved and is a chymotrypsin-like serine protease. It possesses cis-proteolytic activity that cleaves at the W/S scissile bond and separates the capsid protein from the structural polyprotein [32, 36].

The non-human primate sequences possess a stop codon in the nsP3 region, which is associated with enhanced CHIKV replication [3]. Few dissimilarities were noted in two sequences of the Bagan Panchor isolate published in GenBank, FN295483 (MY.37348)-Malaysia-2006 and FN295484 (MY.37350)-Malaysia-2006, i.e., seven amino acid deletions at positions 376–382 and 55V were not found in the four non-human primate isolates or other Bagan Panchor.

### 3.4 Phylogenetic analysis

**Fig. 2:**
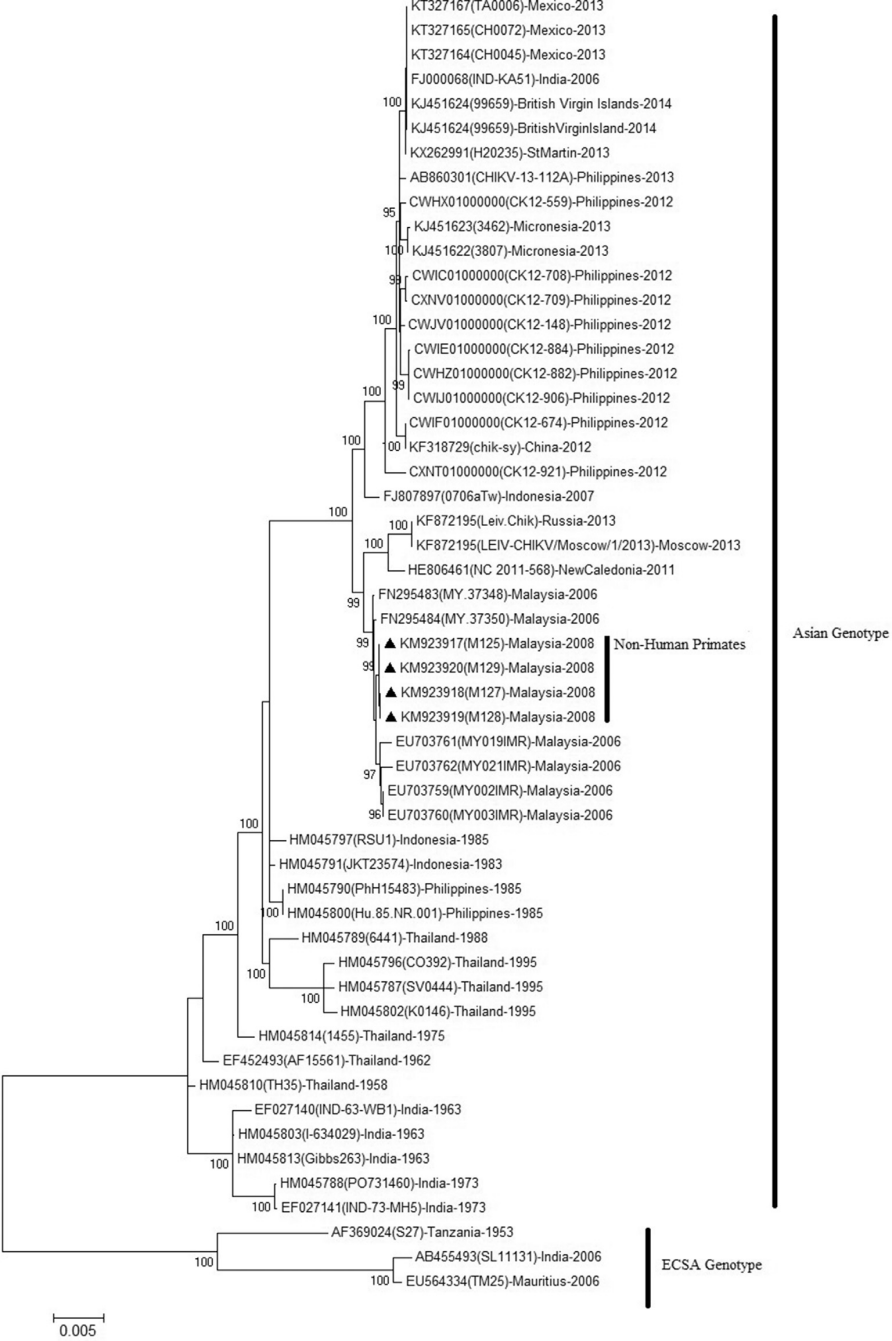
Phylogenetic tree generated by maximum likelihood analysis of the open reading frames (ORFs) (11,238 bp) of CHIKV constructed using MEGA 7.0 software. Four CHIKV isolates from non-human primates are indicated with ▲. Representative strains of each genotype obtained from GenBank are labelled using the following format. “Accession number”, “Isolate”, “Country of origin”, and “Year isolated”. The tree was constructed with 1,000 bootstrap replicates. Bootstrap values (>80%).

**Fig. 3:**
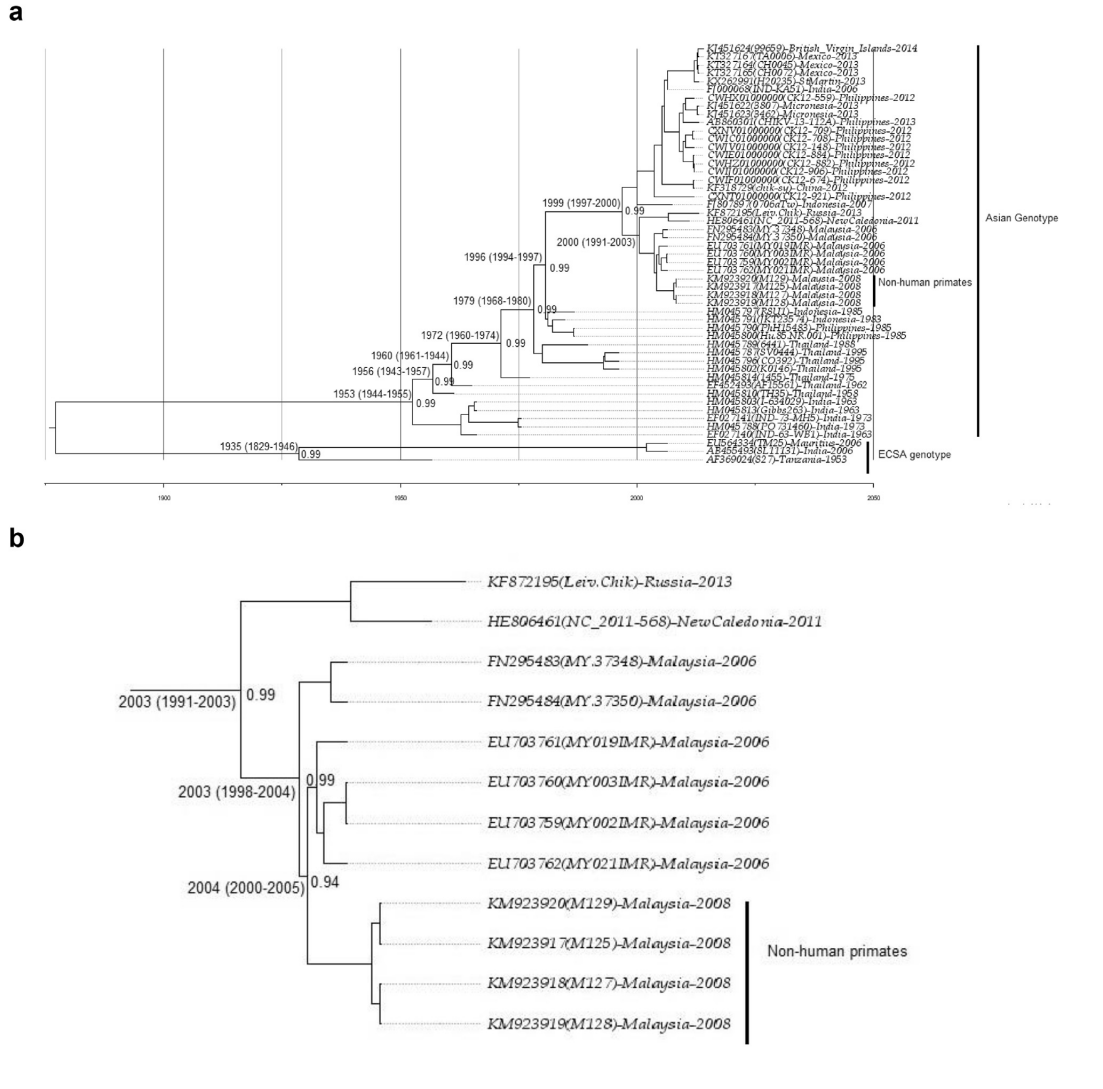
a: Maximum clade credibility (MCC) tree of CHIKV was estimated by Bayesian analysis of 53 open reading frames (ORFs). The posterior probabilities of the key nodes are shown above the respective nodes. The sequences of CHIKV of non-human primates were analysed together with other strains from around the world. The detailed subclades of Malaysian CHIKV are shown in Fig. 3. b: The details of the subclades from Fig. 3a of Malaysian isolates using the maximum clade credibility (MCC) tree of CHIKV were estimated by Bayesian analysis of the open reading frames (ORFs) of Malaysian CHIKV. The posterior probabilities of the key nodes are shown above the respective nodes.

The ML analysis comparing 47 sequences is presented in Fig. 2. The phylogenetic tree showed the two main groups of CHIKV: ECSA and Asian strains (other genotypes were excluded to focus on the Asian genotype linked with our strains). The non-human primate CHIKV isolates clustered together with Asian genotype isolates and were closely related to Bagan Panchor isolates. Bayesian evolutionary analysis elucidates coalescent history. BEAST allowed the use of the isolation year as a calibrating point to estimate the divergence time and then generated a posterior probability (PP) distribution. Phylogenetic tree analysis showed that Malaysian CHIKV isolates possess their own subclade or endemicity, probably Malaysia, which is geographically separated from other countries (Figs. 2 and 3a b). This finding was supported by Sazaly et al. (2007) and Tan et al. (2015) [12,13]. According to Tan et al. (2015) [38], the Asian genotype was separated into two clades, the Indian and Southeast Asian (SEA) lineages, corresponding to distinct spatial and temporal characteristics.

Malaysian CHIKV isolates formed subclades and endemicity with the MRCA estimated in 1991–2003 with the oldest strains in Malaysia (FN295483 and FN295484), which are closely related to those detected in Indonesia [38, 39] (Fig. 3a) and are characterized by seven amino acid deletions (376–382) in the nsP3 region [39]. The sequential amino acid substitution occurred during the evolution of the virus according to specific geographic location or adaptation from Indonesia to Malaysia [38], where all sequences diverged. The divergence of FN295483 and FN295484 according to the MRCA estimation started in Malaysia around 2003 (95% HDP: 1998–2004) (Fig. 3b). Malaysian CHIKV was estimated to start circulating by forming subclades (Fig. 3b) and diverged in humans in the Malaysia region earlier than 2003 (HDP: 1993–2004). This divergence may have started with human encroachment in forest areas for agriculture purposes in rural areas where non-human primates are found, and the MRCA analysis suggested that the initial divergence of CHIKV from human to non-human primates could be dated back to earlier than 2004 (95% HDP 2000–2005). According to the sequence analysis, the vector responsible for transmitting the virus to non-human primates was *Ae. aegypti* because E2-64W, E2-208E, E1- T98, E1-A226, E2-L210, and E2-211I allowed the adaptation of the virus to *Ae. aegypti* [40, 41]. The mean sequence substitution rate of the tree is 2.836 × 10^−4^ substitutions per site per year (95% HPD 2.537 × 10^−4^ to 3.162 × 10^−4)^. Based on this result, the close vicinity of humans and non-human primates may be a contributing factor for human CHIKV spilling over to non-human primates, especially for those that live close to human habitation or vice versa.

The molecular characterization of the full sequence of non-human primate isolates was studied for the first time to provide insight into the origin of CHIKV in non-human primates. Our data showed that the CHIKV in non-human primate infections likely originated from humans, especially from chikungunya outbreaks in Bagan Panchor in 2006, which was in turn related to Klang outbreaks in Selangor in 1998 [37]. The reason behind the appearance of CHIKV in non-human primates is still unknown. However, we propose two possible contributing factors of this occurrence:

First, it is difficult to detect low levels of CHIKV infection in humans. A study in 1960 in Malaysia showed a high CHIKV seropositivity rate in a rural human population. The Malays, who are mainly rural, and the aborigines, who are forest dwellers, had higher frequencies of anti-CHIKV antibodies, which suggested that monkeys could serve as vertebrate hosts of CHIKV in Malaysia [16]. Further studies conducted on Carey Island, situated in the state of Selangor (central western part of Peninsular Malaysia), where monkeys were abundant in mangroves and plantations, showed that plantation workers had anti-CHIKV antibody at fairly high frequencies [16]. Based on a previous study by Marchatte et al. (1978), monkeys may have been one of the CHIKV hosts before our discovery of the virus, but the MCMC phylogeny tree generated using isolation times as calibration points to establish the dates of introduction revealed that CHIKV may have been introduced to non-human primates in 2004. Malaysia experienced the first outbreak CHIKV in late 1998 due to the Asian genotype, involving residents in the suburban area of Klang, a coastal city within the state of Selangor in the central western part of Peninsular Malaysia. Because this was the first occurrence, the diagnosis was confirmed in the laboratory only at the end of January 1999.

Second, our results showed the high similarity of sequences (99.7–99.9%) of human and non-human primate CHIKV, and the minor genetic differences probably facilitated the transmission of the virus to non-human primates because little or no genetic changes are needed by the virus to adapt to the new monkey host [42]. It is therefore possible that non-human primates are infected with human CHIKV, which replicates and uses monkeys as templates or reservoirs. Thus, CHIKV will be maintained zoonotically in the sylvatic environment and will spill over to the human population by infecting the naïve population unexposed to CHIKV, resulting in sporadic outbreaks. In fact, the symptoms and pathology observed in the CHIKV-infected non-human primate model are similar to those previously reported in human CHIKV infection [28].

Our MCC tree showed that the two Malaysian strains FN295483 (MY.37348) and FN295484 (MY.37350) diverged earlier from other Malaysian strains in 2003 (HDP: 1998–2004), as suggested by Sam et al. (2012) [39]. Seven amino acid deletions in FN295483 (MY.37348) and FN295484 (MY.37350) were found, most likely due to the early spread of Asian CHIKV from Indonesia to Malaysia. The deletions in the C-terminal domain of the nsP3 gene may have occurred independently in Malaysia and Indonesia. The C-terminal domain is an important determinative factor in Alphavirus for optimal virus replication in various host cells. This could occur as a result of genetic convergence arising from evolutionary adaptation to local settings [38].

Our study also suggests that CHIKV of non-human primates may infect humans, and such infections are correlated with human mobility. Our findings are important for chikungunya surveillance and may be helpful in developing chikungunya control programmes.

## Declarations

### Author contribution statement

Suhana O.: Conceived and designed the experiments; Performed the experiments; Analyzed and interpreted the data; Wrote the paper.

Nazni W.A.: Conceived and designed the experiments; Contributed reagents, materials, analysis tools or data.

Apandi Y.: Conceived and designed the experiments.

Farah H.: Performed the experiments.

Lee H.L.: Conceived and designed the experiments.

Sofian-Azirun M.: Analyzed and interpreted the data; Wrote the paper.

### Funding statement

This work was supported by the Institute Medical for Research (IMR), Ministry of Health Malaysia, and Malaysia National Institutes of Health Grant (JPP-IMR-07-043).

### Competing interest statement

The authors declare no conflict of interest.

### Additional information

No additional information is available for this paper.
